# Continuing medical education program completion and influencing factors: a cross-sectional study in Sichuan Province, China

**DOI:** 10.3389/fmed.2024.1464039

**Published:** 2024-11-08

**Authors:** Xuedong Liu, Mengliang Ye

**Affiliations:** ^1^Department of Medical Administration, The First People's Hospital of Neijiang, Neijiang, China; ^2^School of Public Health, Chongqing Medical University, Chongqing, China

**Keywords:** continuing medical education, program, completion, influencing factors, China

## Abstract

**Objectives:**

This cross-sectional study was designed to explore the completion of continuing medical education (CME) programs and identify the factors influencing their completion.

**Methods:**

The data for this study were generated from the National CME Program Application and Information Feedback Online System and the Sichuan CME Administrative Platform. The data were processed using descriptive analysis, Chi-square test, and binary logistic regression methods. The completion of each CME program was determined by the research team members according to the criteria created by the Office of the Sichuan CME Commission.

**Results:**

A total of 180 hospitals and 3,622 CME programs were included. Among the 3,622 CME programs, 2,936 (81.1%) were determined to be completed. Comparative analysis showed that in terms of hospital characteristics, specialist hospitals, county hospitals, hospitals with 500–1,000 beds, and hospitals in the regions with government medical expenditure input equal to or more than 3,000 million RMB displayed the highest completion rates. For program attributes, national programs, programs in the field of pharmacy, and programs with 1–3 duration days demonstrated the highest completion rates. The binary logistic regression analysis showed that hospital region with different government medical expenditure input had the strongest positive association with the completion of CME programs [OR = 2.922, 95%CI (1.642–5.198)], while the duration time showed the strongest negative association [OR = 0.235, 95%CI (0.141–0.393)].

**Conclusion:**

This is the first study in China to analyze the completion of CME programs and identify its influencing factors at the provincial level. It is recommended that the government in the region should pay great attention to the construction of measures regarding the factors affecting the completion of CME programs. This includes providing more financial support to CME providers to ensure the formal operation of their CME activities, formulating guidelines on the application of CME programs to reasonably allocate and control the distribution of accredited CME programs across different hospital scales and disciplines, especially offering more training support to county hospitals, promulgating administrative documents to raise attention to the completion of CME programs, and special scrutiny on CME programs with longer durations to provide and protect training opportunities for those in need.

## 1 Introduction

In modern medicine, medical knowledge continues to accelerate, and physicians have a direct obligation to ensure ongoing learning and lifelong education (1) to offer the most up-to-date and highest-quality care to patients. Although the medical profession has grappled with the need for continuous learning for physicians since ancient times, formal credit systems for continuing medical education (CME) began first in North America and subsequently extended to Europe (2) and Asia. With the publication of The Flexner Report on Medical Education in the United States and Canada (3), the then largely unregulated area of postgraduate education known as CME underwent dramatic improvements in the late 1950s and early 1960s (4). At that time, it was widely acknowledged that there was a significant gap between available knowledge and its application in medical practice, and a nationwide plan for CME was regarded as the best solution (5). Several organizations, including the Joint Study Committee, the *Ad Hoc* Committee, and the Advisory Committee on CME (6), were established under the leadership of the American Medical Association to determine the modalities, providers, activities, credit, and accreditation of CME. Since then, CME has been moving toward standardization and practicality.

Over the past few decades, the connotations, attributes, and functions of CME have evolved. Currently, CME aims to update the knowledge, skills, and performance of medical practitioners (7). Along with undergraduate and postgraduate education, CME constitutes the cornerstone of a lifelong learning system (8). Appropriate, adequate, and comprehensive CME is becoming increasingly necessary to maintain professional standards and fulfill the licensing requirements of physicians (9). The implementation of CME involves several imperative segments, such as policy-making, provider and activity accreditation, credit delivery, quality control, and evaluation. Among these, CME providers and activities are the inevitable parts that connect the CME system with healthcare professionals and form the foundation for initially establishing the CME management system. Various CME providers offer diverse CME activities to healthcare professionals worldwide. For instance, in Pakistan, institutions such as medical universities, medical colleges, and medical professional associations have been providing voluntary CME activities for many years (10). Although there are many modalities of CME activities available for healthcare professionals to participate in, such as academic conferences, lectures, government-driven programs, and self-learning (11), large group lecture-based courses still remain one of the most common modalities for CME (12–15). Researchers have studied the effectiveness of the modality of specialized CME programs in enhancing physicians' capabilities to provide high-quality care to certain patients (16–19). The results have shown that evaluating and inspecting these programs can help us better understand the effects and qualities of CME activities and strengthen the management of the CME administrative system.

The establishment of national CME systems in China was compressed into a relatively short period, starting in 1991 but not truly implemented until 2000 (2). In 1991, the then Chinese Ministry of Health (CMH) promulgated “The Continuing Medical Education Interim Regulations,” which officially marked the initiation of national CME practices. In 2000, the CMH revised the existing CME regulations and issued a new regulation entitled “The Continuing Medical Education Regulations (Trial Edition).” Under the guidance of this document, the Chinese National Continuing Medical Education Commission (CCMEC) was established as the official administrative authority for CME to guide, coordinate, and supervise national CME activities. As requested by the CCMEC, the health administrative authorities of 23 provinces, 5 autonomous regions, and 4 municipalities directly under the central government in mainland China have established their own Continuing Medical Education Commissions to formally and uniformly guide the completion of CME activities in their respective regions (20).

CME in China's Sichuan Province officially commenced in 2006 with the promulgation of the administrative document entitled “Standards for the Administration of Continuing Medical Education in Sichuan Province (Trial Edition).” In the same year, the Sichuan Provincial Continuing Medical Education Commission (SCMEC) was established. The SCMEC is responsible for formulating plans and related policies for CME in the region, organizing and conducting CME activities, reviewing and accrediting provincial CME programs, evaluating and recommending national CME programs, examining provincial CME training bases and recommending national CME training bases, awarding and administering Category I credit, supervising remote CME activities, and evaluating and inspecting the effectiveness of CME activities in improving the quality of healthcare provided by medical professionals (21).

The modality of CME activities in Sichuan Province was mainly in the form of programs, and CME programs were mainly divided into five categories: national, provincial, municipal, institutional, and other government promotional programs (22). The providers of national CME programs were limited to medical services, teaching, and research facilities (23), and the providers of provincial CME programs were restricted to health administrative institutions, hospitals, medical associations, universities, and colleges (22). As an inevitable segment of the CME management system, the completion of CME programs by providers directly influences the selection availability and training effects for healthcare professionals. Given its importance, both the CCMEC and SCMEC have decided to strengthen the management of CME providers and programs. In 2019, the CCMEC and SCMEC forwarded the regulation issued by the Chinese National Health Commission entitled “Measures to Alleviate Training Burdens and Advance Continuing Medical Education Works on Grassroots Health Professionals.” It required that CME providers should be accountable for the standard completion of CME programs and that the completion of CME programs by providers will be used as an evaluation criterion during their annual evaluation and inspection (24). These regulations demonstrated that the Chinese CME administrative authorities have given high concerns about the completion of CME programs by providers.

However, despite the magnitude and importance of keeping Chinese physicians in practice up to date, there has been little research on CME programs in China (25). Previous limited studies on CME programs in China have identified several characteristics and attempted to clarify certain issues regarding CME programs. One study aimed to clarify the needs and importance of conducting CME programs in hospitals (26). Some studies explored measures and procedures in hospital management to standardize CME programs and improve quality (27–31). Other studies analyzed the application, accreditation, distribution, completion, and participants of CME programs and proposed measures to strengthen CME management (32–35). However, prior studies were limited to a single institution or a specific urban region. To the best of our knowledge, there have been few studies aiming to analyze CME providers' completion of national and provincial programs at the provincial level and identify factors affecting the completion of CME programs from the perspectives of both CME providers and CME programs.

In light of the aforementioned considerations, our study employed a cross-sectional methodology to elucidate and contrast the completion of provincial and national CME programs in Sichuan Province. Moreover, we sought to identify the underlying factors affecting the completion of CME programs from the perspectives of both CME providers and CME programs. The findings of this study may provide some references for enhancing the management of CME program completion.

## 2 Materials and methods

### 2.1 Study design, setting, and participants

This study was a cross-sectional study and was part of the comprehensive investigation project, “CME Administrative Condition Investigation in Sichuan Province,” which was initiated by the then Health and Family Planning Commission of Sichuan Province in 2017. The providers of hospitals who had participated in the application of national and provincial CME programs during 2015–2017 were included in the study. Led by the Office of SCMEC, a special research team was established to jointly accomplish this study. Although the data for this study may be somewhat outdated, to our knowledge, it was the first and sole study covering the providers of hospitals in national and provincial CME programs at the provincial level. It also analyzed the factors affecting the completion of national and provincial CME programs from both the characteristics of the providers and the programs. Therefore, our data might still be utilized to uncover some potentially valuable results. All members of the research team received training at a training session and conducted the study subsequently from April 21st to April 26th, 2017.

### 2.2 Study material

The material for this study was generated through two steps. Firstly, we ascertained the study providers of CME programs. All providers were classified into five categories: hospitals, centers for disease prevention and control, medical associations, medical colleges, and health administration institutions. We calculated the proportion of each category and found that hospitals accounted for the highest proportion (78.6%) among all accredited CME providers. To efficiently understand the completion of CME providers, the SCMEC office ascertained hospitals as the study providers among the five categories. Secondly, we ascertained the study CME programs. Among the five categories of CME programs, national and provincial CME programs can be awarded category I credit (22), covering almost all disciplines, accounting for an overwhelming proportion of all accredited CME programs, and playing a leading role in China's CME management system. Therefore, they affect almost all healthcare professionals in China. Given this, the SCMEC office decided to select all accredited national and provincial CME programs from 2015–2017 as the study programs.

### 2.3 Data sources and processing

All the data for this study was extracted from the National CME Program Application and Information Feedback Online System (NCPAIFOS) (36) and the Sichuan CME Administrative Platform (SCCAP) (37). Firstly, the filtration word “hospital” was set in the provider column, allowing the systems to generate the study hospitals. Secondly, all the accredited national and provincial CME programs of the study hospitals were included as the study programs. This resulted in the final sample size of 180 study hospitals and 3,622 study programs. For further analysis, we matched and extracted certain characteristics of hospitals and programs. Firstly, according to the administrative documents entitled “Regulations on the Administration of Medical Institutions in Sichuan Province” promulgated by the Health Commission of Sichuan Province (38), “Sichuan Statistical Yearbook (2015–2017)” issued by the Sichuan Provincial Bureau of Statistics (39), and the information on the official website of each hospital, the study hospitals were matched to different categories, types, levels, ownership forms, scales, and regions with different government medical expenditure inputs. Secondly, ten characteristics of the study programs were extracted, including the number, title, provider, applicant, type, category, accredited year, discipline, duration time, and the number of credits awarded. Thirdly, the feedback materials of the study programs were extracted, including completion demonstration document, completion arrangement, training pictures, the signature sheet of participants, training textbooks, conclusion document, and a completion feedback chart. These were extracted to determine whether the program was completed or not.

### 2.4 Study program completion determination

**Figure 1:**
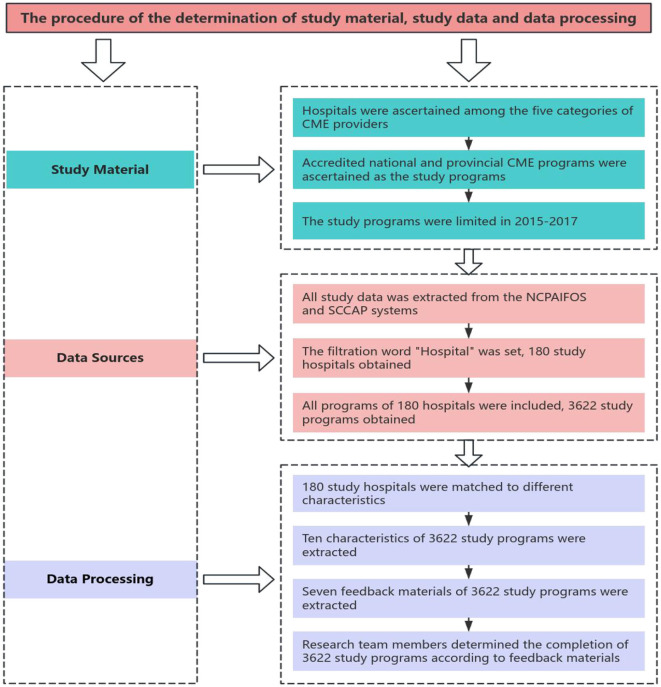
The flow chart of the determination of study material, study data, and data processing.

The SCMEC office stipulated that all seven feedback materials uploaded to NCPAIFOS for national CME programs and SCCAP for provincial CME programs must be in compliance with the regulated time and in complete and standard forms before it could be assessed as completed (22). To determine the completion of 3,622 study programs, each member of the research team was assigned a specific number of programs to manually determine the completion of each program according to the criterion before December 31st, 2017. The detailed procedure for data sources, processing, and program completion determination is illustrated in Figure 1.

### 2.5 Statistical analysis

The data analysis was conducted using SPSS (version 26.0). After the screening process, variables such as the providers' category, type, level, ownership form, scale, region with different government medical expenditure input, and variables associated with the programs' type, category, accredited year, discipline, duration time, and number of credits awarded were included for statistical analysis. To ensure the feasibility of the analysis, all variables were converted into categorical variables and presented in the form of counts and proportions.

Firstly, we applied descriptive analysis to understand the distribution of study providers and study programs in terms of their characteristics. Secondly, a Chi-square test was employed to compare differences between different characteristics of the study providers and the study programs in the rates of CME program completion. Thirdly, a binary logistic regression model was constructed to identify the influencing factors related to the completion of study programs from both the characteristics of providers and programs. All characteristics of study providers and study programs were included as independent variables. The completion of study programs was set as the dependent variable. The odds ratio (OR), 95% confidence interval (CI) for OR, and *P* values were reported when conducting logistic regression analysis. In this study, a *P* value < 0.05 was considered statistically significant.

## 3 Results

### 3.1 The characteristics of the CME providers and programs

With regard to CME providers, a total of 180 hospitals have been identified as providing national and provincial CME services in Sichuan Province from 2015 to 2017. Among these, the majority were municipal hospitals, accounting for 46.1%. Additionally, 64.4% were general hospitals, 80.6% were tertiary hospitals, and 90.0% were public hospitals. The overwhelming hospitals (32.8%) had 500–1,000 beds and were located in the regions with government medical expenditure input equal to or greater than 6,000 million RMB (42.2%). With respect to CME programs, a total of 3,622 accredited national and provincial CME programs were extracted for study. Of these, the vast majority were provincial programs, accounting for 73.7%. Furthermore, 92.4% were newly applied programs, 39.2% were accredited in 2017, 65.0% belonged to the discipline of medicine, 56.4% had the duration time of 3–5 days, 50.5% awarded 2–4 credits. The detailed distribution of the characteristics of CME providers and programs is shown in Table 1.

**Table 1:** The distribution of the characteristics of CME providers and programs.

**Characteristics of providers**	**Count**	**Percent**	**Characteristics of programs**	**Count**	**Percent**
**Hospital type**			**Program type**		
General hospital	116	64.4	Provincial program	2,669	73.7
Specialist hospital	64	35.6	National program	953	26.3
**Hospital category**			**Accredited year**		
Provincial hospital	23	12.8	In 2017	1,417	39.2
Municipal hospital	83	46.1	In 2016	1,199	33.1
County hospital	74	41.1	In 2015	1,006	27.7
**Hospital scale (bed number)**			**Program discipline**		
< 500	41	22.8	Medicine	2,355	65.0
500–1,000	59	32.8	Nursing	620	17.1
1,000–1,500	35	19.4	Public health	235	6.5
1,500–2,000	24	13.3	Medical technology	344	9.5
≥2,000	21	11.7	Pharmacy	68	1.9
**Hospital ownership form**			**Program category**		
Public hospital	162	90.0	Newly applied program	3,346	92.4
Private hospital	18	10.0	Recorded program	276	7.6
**Hospital level**			**Duration time**		
Tertiary hospital	145	80.6	1~	837	23.1
Secondary hospital	31	17.2	3~	2,041	56.4
Primary hospital	4	2.2	5~	668	18.4
**Hospital regions with different government medical expenditure input** **(million, RMB)**			7~	76	2.1
< 1,500	8	4.4	**Number of credits awarded**		
1,500–3,000	51	28.3	2~	1,828	50.5
3,000–4,500	28	15.6	4~	827	22.8
4,500–6,000	17	9.4	6~	428	11.8
≥6,000	76	42.2	8~	539	14.9

### 3.2 The differences in the completion of the CME programs

Among the 3,622 CME programs, 2,936 (81.1%) were assessed as completed by research team, while 686 (18.9%) were determined as uncompleted. Comparative analysis showed that, for hospital characteristics, the lowest completion rate was observed in general hospitals (79.2%), municipal hospitals (79.8%), hospitals with equal to and more than 2,000 beds (74.7%), and hospitals in the regions with government medical expenditure input between 1,500–3,000 million RMB (72.1%). In contrast, the highest completion rate was observed in specialist hospitals (86.8%), county hospitals (86.4%), hospitals with 500–1,000 beds (87.2%), and hospitals in the regions with government medical expenditure input between 4,500–6,000 million RMB (85.9%). For program characteristics, provincial programs (80.2%), programs in the field of medicine (79.8%), and programs with equal to or more than 7 duration days (59.2%) showed the lowest completion rate. Conversely, national programs (83.5%), programs in the field of pharmacy (86.8%), and programs with 1–3 duration days (83.5%) demonstrated the highest completion rate. The detailed results of the comparative analysis are presented in Table 2.

**Table 2:** The comparison of program completion among different characteristics of providers and programs.

**Characteristics of providers**	**Completion condition**		***χ^2^***	***P***	**Characteristics of programs**	**Completion condition**		***χ^2^***	***P***
	**Un-Completed**	**Completed**				**Un-Completed**	**Completed**		
**Hospital type**			25.459	**< 0.001**	**Program type**			5.121	**0.024**
General hospital	569 (20.8)	2,165 (79.2)			Provincial program	529 (19.8)	2,140 (80.2)		
Specialist hospital	117 (13.2)	771 (86.8)			National program	157 (16.5)	796 (83.5)		
**Hospital category**			11.715	**0.003**	**Accredited year**			2.988	0.224
Provincial hospital	264 (19.3)	1,101 (80.7)			In 2017	252 (17.8)	1,165 (82.2)		
Municipal hospital	351 (20.2)	1,384 (79.8)			In 2016	227 (18.9)	972 (81.1)		
County hospital	71 (13.6)	451 (86.4)			In 2015	207 (20.6)	799 (79.4)		
**Hospital scale**			79.625	**< 0.001**	**Program discipline**			10.107	**0.039**
< 500	32 (18.9)	137 (81.1)			Medicine	475 (20.2)	1,880 (79.8)		
500–1,000	62 (12.8)	422 (87.2)			Nursing	93 (15.0)	527 (85.0)		
1,000–1,500	71 (13.7)	448 (86.3)			Public health	43 (18.3)	192 (81.7)		
1,500–2,000	116 (13.6)	736 (86.4)			Medical technology	66 (19.2)	278 (80.8)		
≥2,000	405 (25.3)	1,193 (74.7)			Pharmacy	9 (13.2)	59 (86.8)		
**Hospital ownership form**			2.358	0.125	**Program category**			0.467	0.495
Public hospital	673 (18.8)	2,902 (81.2)			Newly applied program	638 (19.1)	2,708 (80.9)		
Private hospital	13 (27.7)	34 (72.3)			Recorded program	48 (17.4)	228 (82.6)		
**Hospital level**			0.581	0.612	**Duration time**			37.472	**< 0.001**
Tertiary hospital	666 (18.8)	2,870 (81.2)			1~	138 (16.5)	699 (83.5)		
Secondary hospital	18 (23.1)	60 (76.9)			3~	361 (17.7)	1,680 (82.3)		
Primary hospital	2 (25.0)	6 (75.0)			5~	156 (23.4)	512 (76.6)		
**Hospital regions with different government medical expenditure input (million, RMB)**			41.820	**< 0.001**	7~	31 (40.8)	45 (59.2)		
< 1,500	26 (25.5)	76 (74.5)			**Number of credits awarded**			5.898	0.117
1,500–3,000	155 (27.9)	400 (72.1)			2~	324 (17.7)	1,504 (82.3)		
3,000–4,500	117 (16.1)	609 (83.9)			4~	178 (21.5)	649 (78.5)		
4,500–6,000	41 (14.1)	249 (85.9)			6~	77 (18.0)	351 (82.0)		
≥6,000	347 (17.8)	1,602 (82.2)			8~	107 (19.9)	432 (80.1)		

Data are presented as *n* (%). P value is less than 0.05, indicating the result is statistically significant.

### 3.3 The influencing factors affecting the completion of the CME programs

A total of 12 independent variables were selected to construct the binary logistic regression model, with the completion of the CME programs being designated as the dependent variable. The independent variables were incorporated into the model using the “Forward Likelihood Ratio” method. The results of the binary logistic regression analysis (Table 3) demonstrated that regarding the probabilities of the completion of CME programs, hospitals with 1,000–1,500 beds exhibited a 1.763-fold higher probability than those with < 500 beds (OR = 1.763), while hospitals with equal to or above 2,000 beds displayed a 1.587-fold lower probability than those with < 500 beds (OR = 0.630). Compared to hospitals in the < 1,500 million RMB government medical expenditure input regions, hospitals with equal to or above 3,000 million RMB regions all showed an increase of 2.638 times (OR,=,2.638), 2.922 times (OR = 2.922), and 1.643 times (OR,=,1.643), respectively. Additionally, provincial programs exhibited a 1.567-fold reduction compared to national programs (OR = 0.638). Programs in the field of nursing exhibited a 1.698-fold higher probability than those in the field of medicine (OR = 1.698). Compared to programs with 1–3 duration days, those with longer duration days presented significantly lower probabilities of being completed. The probabilities were 1.342 times lower (OR = 0.745), 2.160 times lower (OR = 0.463), and 4.255 times lower (OR = 0.235), respectively.

**Table 3:** Logistic regression analysis on the factors affecting the completion of the CME programs.

**Variables**	***B***	**SE**	**P**	**OR**	**95% CI for OR**	
					**Lower**	**Upper**
**Hospital scale** **(compared with** ** < 500 beds)**			0.000			
With 500–1,000 beds	0.434	0.244	0.075	1.544	0.957	2.491
With 1,000–1,500 beds	0.567	0.240	**0.018**	1.763	1.101	2.823
With 1,500–2,000 beds	0.388	0.225	0.085	1.474	0.948	2.293
With equal to or above 2,000 beds	−0.462	0.212	**0.029**	0.630	0.416	0.954
**Hospital regions with different government medical expenditure input** **(compared with** ** < 1,500 million RMB)**			0.000			
1,500–3,000 million RMB	−0.122	0.252	0.628	0.885	0.540	1.451
3,000–4,500 million RMB	0.970	0.259	**0.000**	2.638	1.587	4.386
4,500–6,000 million RMB	1.072	0.294	**0.000**	2.922	1.642	5.198
≥6,000 million RMB	0.496	0.246	**0.043**	1.643	1.015	2.659
**Program type**	−0.450	0.113	**0.000**	0.638	0.511	0.796
**Program discipline** **(compared with medicine discipline)**			0.002			
Pharmacy	0.287	0.372	0.440	1.333	0.643	2.761
Nursing	0.53	0.13	**0.000**	1.698	1.315	2.193
Medical technology	0.165	0.152	0.277	1.179	0.876	1.588
Public health	0.006	0.183	0.975	1.006	0.702	1.44
**Duration time** **(compared with 1**–**3 duration days)**			**0.000**			
3~	−0.294	0.115	**0.011**	0.745	0.594	0.935
5~	−0.769	0.146	**0.000**	0.463	0.348	0.617
7~	−1.448	0.262	**0.000**	0.235	0.141	0.393

P value is less than 0.05, indicating the result is statistically significant.

## 4 Discussion

Since 2019, the National Health Commission of China has put forward certain regulations for CME providers to strengthen the management of CME activities (23). In accordance with these regulations, the office of CCMEC promulgated a specific document entitled “Continuing Medical Education Evaluation Indexes on Healthcare Institutions.” This document established a 100 percent completion rate for CME programs for each CME provider for the first time (40). To meet the CME management requirements of administrative authorities, it is crucial to figure out the completion conditions of CME programs and identify the factors that affect the further enhancement of CME management. The management of CME programs is a systematic and closed-loop process. Some researchers have proposed integrating the life-cycle theory into the management practice of CME programs. They suggested regarding CME programs as a living entity and constructing a seamless management model, encompassing all aspects of the program, including application, accreditation, publication, planning, dissemination, completion, inspection, and feedback (41). This theory may be applied to the modern management of CME programs. In this study, the most critical point of the completion of CME programs and the affecting factors were examined through the statistical approach and the logistic model established on the characteristics of CME providers and programs. To the best of our knowledge, some novel findings on the completion of CME programs have been generated for the first time.

### 4.1 Overall completion of the study CME programs

The study revealed that the overall completion rate of national and provincial CME programs in Sichuan Province between 2015 and 2017 was 81.1 percent, which was lower than the 100 percent requirement set by the CCMEC (40). Over the past few decades, the scale of accredited CME programs has expanded exponentially due to the increasing CME needs of healthcare professionals (42). However, the completion of CME programs do not match the rapid expansion of accredited CME programs, as the completion rate remains relatively low. Possible explanations for the low completion rate of CME programs are as follows. Currently, the predominant management model for CME programs in China is accreditation and completion management. Regarding accreditation management, CME administrative authorities mainly focus on examining the modalities of application materials, including the credentials of CME providers, applicants, training teachers, and the comprehensiveness and suitability of contents. Expert assessments from professional perspectives on the applying CME programs are scarce and only exist in a few regions due to the difficulties in organization and budget shortages. This may lower the access threshold for obtaining the accreditation qualification and decrease the attention of applicants. With respect to completion management, the constraints of human resources and budgets make it challenging for CME administrative authorities to implement a comprehensive on-site assessment of all programs. Moreover, the lack of real-time monitoring mechanisms and systems for the management of CME programs at all levels further deteriorates the availability and feasibility of conducting a comprehensive inspection on the completion of CME programs (43). Consequently, the completion of CME programs is somewhat decentralized, spontaneous, and chaotic (43, 44). These findings are consistent with those of previous studies on the completion rate of CME programs (33, 45). It is recommended that CME administrative authorities in the region should firstly shift their awareness on CME program management. Instead of focusing on the expansion of accredited number of CME programs, their attention should be directed toward playing full utilization of the existing accredited programs (45). A potential solution is to introduce the process management theory into the management of CME programs. Specially, the management of CME programs is divided into three stages namely application, completion, and feedback management. In the application stage, an expert team should be established to evaluate the applying programs' “four news” (new theories, new technologies, new methods, new knowledge) and “three characteristics” (pertinence, practicability, and advancement). Only those meet these standards could be identified as accredited programs. In the completion stage, multiple techniques can be adopted to monitor the completion conditions of CME programs such as random on-site inspections and online real-time monitoring through mobile applications. In the final stage, significant focuses should be given to monitor and evaluate the feedback materials upload conditions, including its punctuality, comprehensiveness, and standardization, to holistically understand the training effects of each program and make the timely corresponding enhancements (33).

### 4.2 Differences in the completion of the study CME programs

The study observed significant differences in the completion of the study CME programs from the perspectives of both the characteristics of providers and programs. Researchers have identified gaps in the planning of CME program. They reported that to secure the training effectiveness of CME programs, it was essential to adhere to the fundamentals of practical training, which include planning and developing an effective program, proper completion, and effective evaluation of the teaching and learning process (46). The identification of the differences in the completion of CME programs may inform the development of corresponding measures to enhance the proper completion of these programs. With regard to the providers of CME programs, our study found that general hospitals and hospitals of the largest scale presented the lowest completion rate of CME programs, while county hospitals displayed the highest completion rate. General hospitals and hospitals of the largest scale accounted for the predominant number of accredited CME programs. They are commonly the most powerful and influential hospitals in the region. Consequently, they have a favorable chance of having their programs successfully accredited, thus accounting for the overwhelming proportion of all the accredited programs. However, due to the massive burden of a large number of programs, heavy clinical workloads, and the conventional view of “positive application, optional or negative completion” (44), the applicants from general hospitals and hospitals of the largest scale may neglect the importance of the completion of the accredited CME programs. This has led to many programs being wasted and not being fully utilized to meet the needs of healthcare professionals. County hospitals occupied only 14.4% of all the accredited CME programs, far below the ratio 47.9% of municipal hospitals and 37.6% of provincial hospitals. This is consistent with the previous finding that the applicants of CME programs in western region, China, are concentrated within the scope of tertiary grade A hospitals (47). County hospitals are more vulnerable to poor medical techniques, lower hospital reputation, and low competitiveness of healthcare professionals when competing with upper-level hospitals in the application process of CME programs. As a result, they have the least possibility of having their programs approved and obtain a limited number of accredited programs. However, healthcare professionals in rural areas in 11 western provinces in China had a strong need for CME (48). Facing the confounding situations of the limited number of accredited CME programs and the huge training needs of healthcare professionals, county hospitals may be most concerned with the prompt and formative completion of programs and display the highest completion rate compared to other hospitals. Inspired by the finding that there is strong support for industry support of CME in China (25), we constructively incorporated the concept of government medical expenditure input into the comparison of the completion of CME programs. The results demonstrated that hospitals in the higher government medical expenditure input regions displayed higher completion rates of CME programs. Hospitals in the higher government medical expenditure regions may acquire higher financial support to offset their operating costs. Therefore, they are more willing to invest more resources to support and even award the completion of CME programs (45). This may improve the applicants' enthusiasm to apply for and complete CME programs and guarantee a higher completion rate than other hospitals in the lower government medical expenditure input regions.

With regard to the characteristics of CME programs, provincial programs, programs in the field of medicine, and programs with seven or above duration days showed significantly the lowest completion rate. Possible explanations may be as follows. A prior study has pointed out that national programs received more attention than provincial and municipal programs in both application and completion (33). The national CME programs are better authoritative demonstrations of providers' comprehensive strengths and are the intermediaries to enlarge the providers' academic reputations. Therefore, the providers of national programs are more willing to promulgate awarding documents to boost the completion of national programs. Programs in the field of medicine have the highest number among all the accredited programs. The distribution of accredited CME programs in Sichuan province is mainly concentrated in the field of medicine, which is consistent with a previous study on the further standardization and perfection of CME in China (43). The inclination of CME programs toward medicine may lower the difficulty in applying for new CME programs in this field and increase the possibility of successful accreditation. However, this may reduce the attention of CME applicants in this field to the completion of accredited programs and ultimately lower the completion rate. Programs with longer duration days commonly require more expenditures and greater human resources and energy to organize, control, and conduct. When unplanned events occur, the applicants may switch to easy programs with shorter duration days to meet the lowest completion rate requirements set by the local CME administrative authorities. This may also decrease the completion rate of programs with longer duration days.

### 4.3 Factors affecting the completion of the study CME programs

A total of five factors have been identified that could potentially affect the completion of CME programs. The strongest positive association was observed between hospital regions with different government medical expenditure inputs and the completion of CME programs. The finding illustrated that hospitals in regions with higher government medical expenditure inputs all displayed higher probabilities of completion of the accredited CME programs compared to those in the lowest regions. Higher government medical expenditure input may lead to a reduction in the burden on hospitals to conduct formal CME activities, thus facilitating the development of CME in the region. However, researchers have discovered that the majority of hospitals in China have not received educational investment from the government and have instead borne the costs themselves or through healthcare professionals (44, 49). Consequently, researchers have discovered that Chinese CME participants preferred industry-supported CME and strongly favored industry offsetting costs, regardless of age, academic rank, or practice type, even though most of them believed that industry support created bias in CME (25). This was evidenced by the relevant studies aiming to clarify the influences of industry-authored educational materials on continuing education activities. They have discovered that industry-authored educational materials might have promotional intent and unintended, negative consequences for conducting continuing education (50, 51). In the era of modern education, government investment on education should be the primary form of education investment (49). To safeguard the formal operation of CME, it is recommended that the government should balance financial support in the region, especially in those rural regions, to guarantee the completion of CME programs from the impacts of the industry and to increase the overall completion rate. In contrast, the duration days of CME programs demonstrated the strongest negative association with the completion of CME programs. The results confirmed that CME programs with longer duration days displayed lower probabilities of completion of CME programs. This may be related to both CME participants and applicants. In China, a national mandate requires healthcare professionals to acquire at least 25 credits per year (2). Healthcare professionals must select a specific number of programs or even irrelevant programs to fulfill the credit requirement (52). However, they are typically occupied by daily work routines and have limited time to participate in CME training. Therefore, time is a crucial factor in their selection of CME programs to attend (18). Due to time limitations, they commonly select programs with higher credits to quickly fulfill the requirement, which leads to higher demands on programs with higher credits. As for CME applicants, they may also be inclined to apply for programs with longer duration days to attract more participants and expand the impact of the programs, as they can provide more credits to participants. However, programs with longer duration days demand more expenditure due to the increased number of staff and resources. In addition, the period between the submission of an application and the completion of a CME program is relatively long (53), and the number of teachers required for programs with longer duration days is greater than those with shorter duration days. Due to unforeseen circumstances, the original plans for programs such as teachers and teaching hours are frequently altered, making it challenging to complete the CME programs as intended. Furthermore, applicants for national and provincial CME programs in China are required to be healthcare professionals with senior titles (22, 23). Such individuals are often engaged in substantial clinical and administrative duties, making it challenging for them to ascertain the efficacy of their accredited programs, let alone take responsibility for organizing and completing them. Programs with longer duration days may significantly increase the workload and training pressure on those involved in the completion of such programs. When the arrangements for the completion of CME programs conflict with their daily schedules, they may be unable to complete the accredited programs.

Several limitations of our study should be noted. Firstly, the study was conducted in 2017. This means that the data for this study are somewhat outdated and cannot provide the most recent findings. Secondly, the data were generated from the NCPAIFOS and the SCCAP systems, which specifically represent the completion conditions of Sichuan Province, China. Therefore, the data bias should be taken into account when the findings of this study are generalized to other regions. Thirdly, although all members of the research team were trained to use the same criteria to judge the completion of CME programs, there were still some discrepancies among different research members. As a result, the findings of this study may slightly deviate from the genuine practices. Fourth, as this was a cross-sectional study, a causal relationship between the characteristics of CME providers and CME programs and the completion of CME programs could not be concluded. The influencing factors will change over time. Therefore, further studies with updated variables on the completion and influencing factors of CME programs are still recommended.

## 5 Conclusion

To the best of our knowledge, this is the first study in China to analyze the completion and identify the factors affecting the completion of national and provincial CME programs at the provincial level. The results demonstrated that the overall completion rate of national and provincial CME programs remained relatively low. For hospital characteristics, specialist hospitals, county hospitals, hospitals with 500–1,000 beds, and hospitals in the regions with government medical expenditure input equal to or more than 3,000 million RMB displayed the highest completion rate. For program attributes, national programs, programs in the field of pharmacy, and programs with 1–3 duration days demonstrated the highest completion rate. Hospital region with different government medical expenditure input showed the strongest positive association with the completion of CME programs, while the duration time showed the strongest negative association. The completion of national and provincial CME programs in Sichuan Province, China, should be further improved. It is recommended that the government in the region should pay great attention to constructing measures on the factors affecting the completion of CME programs. This includes providing more financial support to CME providers to ensure the formal operation of their CME activities, formulating guidelines on the application of CME programs to reasonably allocate and control the distribution of accredited CME programs across different hospital scales and disciplines, especially more training support to county hospitals, promulgating administrative documents to raise attention to the completion of CME programs, and special scrutiny on CME programs with longer duration days to provide and protect training opportunities for those in need.

## Data Availability

The raw data supporting the conclusions of this article will be made available by the authors, without undue reservation.

## References

[1] Nissen SE. Reforming the continuing medical education system. JAMA. (2020) 313:1813–4. 10.1001/jama.2015.4138 25965221

[2] Miller LA Chen XJ Srivastava V. CME credit systems in three developing countries: China, India and Indonesia. JECME. (2015) 4:27411. 10.3402/jecme.v4.27411

[3] Flexner A. The Flexner Report on Medical Education in the United States and Canada 1910. Bethesda (MD): Science and Health Publications, Inc (1910). 10.1126/science.32.810.41 17745880

[4] Ruhe CH. The American medical association's program of accreditation in continuing medical education. J Med Educ. (1964) 39:670–8. 14154511

[5] Dryer BV. Lifetime learning for physicians. Principles, practices, proposals. J Med Educ. (1962) 37:1–134. 13887979

[6] Kokemueller P Osguthorpe JD. Trends and developments in continuing medical education. Otolaryngol Clin N Am. (2007) 40:1331–45. 10.1016/j.otc.2007.08.003 18021844

[7] Bloom BS. Effects of continuing medical education on improving physician clinical care and patient health: a review of systematic reviews. Int J Technol Assess Health Care. (2005) 21:380–5. 10.1017/S026646230505049X 16110718

[8] Hilton SR Slotnick HB. Proto-professionalism: how professionalisation occurs across the continuum of medical education. Med Educ. (2005) 39:58–65. 10.1111/j.1365-2929.2004.02033.x 15612901

[9] Gu J Zhu S Chen T Tang J Pan Z Gong J. Evaluation of the Spring Seedling Project—Zhaotong Program: a study of a novel continuing medical education program for rural doctors in China. AJRH. (2020) 28:434–42. 10.1111/ajr.12659 32985023 PMC7756282

[10] Vakani FS Demirkol A Uebel K Balasooriya C. CME providers' experiences and practices in Pakistan: a case study. BMC Med Educ. (2024) 24:272. 10.1186/s12909-024-05201-y 38475806 PMC10935942

[11] Aparicio A Chaudhry HJ Staz M Cain F Mayo WS Karty A. Supporting physician lifelong learning through effective continuing medical education and professional development. J Med Regul. (2016) 102:7–15. 10.30770/2572-1852-102.1.727754500

[12] Yee M Simpson-Young V Paton R Zuo Y. How do GPs want to learn in the digital era? Aust Fam Physician. (2014) 43:399–402. 24897992

[13] Armstrong E Parsa-Parsi R. How can physicians' learning styles drive educational planning? Acad Med. (2005) 80:680–4. 10.1097/00001888-200507000-00013 15980086

[14] Stancic N Mullen PD Prokhorov AV Frankowski RF McAlister AL. Continuing medical education: what delivery format do physicians prefer? J Contin Educ Health Prof . (2003) 23:162–167. 10.1002/chp.1340230307 14528787

[15] Copeland HL Longworth DL Hewson MG Stoller JK. Successful lecturing: a prospective study to validate attributes of the effective medical lecture. J Gen Intern Med. (2000) 15:366–371. 10.1046/j.1525-1497.2000.06439.x 10886470 PMC1495460

[16] Casey A-N Islam MM Schütze H Parkinson A Yen L Shell A. GP awareness, practice, knowledge and confidence: evaluation of the first nation-wide dementia-focused continuing medical education program in Australia. BMC Fam Pract. (2020) 21:1–16. 10.1186/s12875-020-01178-x 32522153 PMC7285709

[17] Fisher WA Gilca V Murti M Orth A Garfield H Roumeliotis P. Continuing medical education improves physician communication skills and increases likelihood of pediatric vaccination: findings from the pediatric influenza vaccination optimization trial (PIVOT)-II. Vaccines. (2023) 11:1–10. 10.3390/vaccines11010017 36679861 PMC9861912

[18] Schütze H Shell A Brodaty H. Development, implementation and evaluation of Australia's first national continuing medical education program for the timely diagnosis and management of dementia in general practice. BMC Med Educ. (2018) 18: 194. 10.1186/s12909-018-1295-y 30097036 PMC6086051

[19] Liang L-B Li X Liu X-P Li C-Z Luo D Liu F. Evaluation of the star family doctors training program: an observational cohort study of a novel continuing medical education program for general practitioners within a compact medical consortium: a quantitative analysis. BMC Med Educ. (2023) 23:250. 10.1186/s12909-023-04210-7 37069532 PMC10108467

[20] Ministry of Health, Ministry of Personnel. The Continuing Medical Education Regulations (Trial Edition). Available at: https://www.med66.com/jixuyixuejiaoyuwang/jixuyixuejiaoyuxueshuhuiyi/cy1908285491.shtml (accessed October 22, 2024).

[21] Health Bureau of Sichuan Province. Standards for the Administration of Continuing Medical Education in Sichuan Province (Trial Edition). Available at: https://www.med66.com/new/14 18a1439aa2009/20091018zhangf154820.shtml (accessed October 22, 2024).

[22] Health Commission of Sichuan Province. Measures for the Management of Continuing Medical Education Programs in Sichuan Province (trial edition). Available at: http://wsjkw.sc.gov.cn/scwsjkw/csgz/2017/9/11/db9a9aa5eb7e4ff8aa80a9df8b4237d5.shtml (accessed October 22, 2024).

[23] Office of CCMEC. Methods for the application and recognition of national continuing medical education program. Available at: https://www.cma.org.cn/col/col8/index.html (accessed October 22, 2024).

[24] National Health Commission of China. Measures to alleviate training burdens and advance continuing medical education works on grassroots healthcare professionals. Available at: http://www.nhc.gov.cn/qjjys/s7949/201909/25cdfa6c2a51446eb79e50eb7ca0b9ee.shtml (accessed October 22, 2024).

[25] Stephenson CR Qian Q Mueller PS Schleck CD Mandrekar JN Beckman TJ. Chinese physician perceptions regarding industry support of continuing medical education programs: a cross-sectional survey. Medical Educ Online. (2020) 25:1694308. 10.1080/10872981.2019.1694308 31747854 PMC6882482

[26] Hua B. The need and significance of continuing medical education programs in hospitals (in Chinese). Continuing Medical Educ. (2020) 34:1–2. 10.3969/j.issn.1004-6763.2020.07.001

[27] Hongmei D Min M Yan W. A preliminary study on the standardized management practice of continuing medical education projects (in Chinese). Continuing Medical Educ. (2020) 34:1–2. 10.3969/j.issn.1004-6763.2020.03.001

[28] Fan S Mingjie F Huaiwei H. A brief talk on the current situation of the management of continuing medical education projects and its countermeasures (in Chinese). Chinese Continuing Medical Educ. (2020) 12:1–2. 10.3969/j.issn.1674-9308.2020.01.001

[29] Cuiwei L Lin Y. Promoting process management and quality assurance of national continuing medical education programs with new technologies (in Chinese). China Continuing Medical Educ. (2020) 13:1–6. 10.3969/j.issn.1674-9308.2021.34.001

[30] Xiaoxiao Z Juan L Qingqi Z. Study on influencing factors of the national continuing medical education projects' quality (in Chinese). China Higher Med Educ. (2019) 34:1–2. 10.3969/j.issn.1002-1701.2019.10.001

[31] Cheng X Guizhi Z Lin C. Practice and innovation of standardized quality management of continuing medical education programs in Chinese Medicine (in Chinese). Zhejiang Chinese Med. (2023) 58:464–5. 10.3969/j.issn.0411-8421.2023.06.039

[32] Collaborators G2TF. Analysis on the management of continuing medical education projects in 6 hospitals in Yangzhou (in Chinese). Modern Hospitals. (2019) 19:1736–8. 10.3969/j.issn.1671-332X.2019.12.00639366729

[33] Xin Z. Analysis of the current status of continuing medical education programs in a specialist hospital over the past five years (in Chinese). Continuing Medical Educ. (2023) 37:101–4. 10.3969/j.issn.1004-6763.2023.06.026

[34] Jin D Lv S Shanshan L Xingqiang H. A brief analysis on the continuing medical education projects of Anhui Provincial Center for Disease Control and Prevention from 2015 to 2019 (in Chinese). China Continuing Medical Educ. (2021) 13:57–60. 10.3969/j.issn.1674-9308.2021.17.016

[35] Na Z Jinfeng Z Yu L Li D. Analysis on continuing medical education conduction and exploration on teaching management mode (in Chinese). Modern Hospitals. (2021) 21:1442–4. 10.3969/j.issn.1671-332X.2021.09.039

[36] Chinese Medical Association. National CME Program Application and Information Feedback Online System. Available at: https://cmegsb.cma.org.cn/national_project/login.jsp (accessed May 14, 2024).

[37] Sichuan Provincial Health Information Center. Administrative Management Platform for Continuing Medical Education Program of Sichuan Province. Available at: http://202.61.90.26:9000/#/ (accessed May 14, 2024).

[38] Health Commission of Sichuan Province. Regulations on the Administration of Medical Institutions in Sichuan Province. Available at: https://wsjkw.sc.gov.cn/scwsjkw/fg1/2022/12/22/625ea742 9caf49b0a3e77ee579229bc6.shtml (accessed October 22, 2024).

[39] Sichuan Provincial Bureau of Statistics. Sichuan Statistical Yearbook (2015-2017). Available at: https://tjj.sc.gov.cn/scstjj/c105855/nj.shtml (accessed May 14, 2024).

[40] Office of CCMEC. Continuing Medical Education Evaluation Indexes on Healthcare Institutions. Available at: https://www.cma.org.cn/art/2021/10/18/art_91_41631.html (accessed October 14, 2021).

[41] Zhen M Wei H Jing H Hui J. Application of life cycle management in national continuing medical education project management (in Chinese). Chin J Med Edu Res. (2017) 16:204–8. 10.3760/cma.j.issn.2095-1485.2017.02.02430704229

[42] Zhen M Qi Z Meili D Wei H Jing H Hui J. Practice and reform of whole process management of national medical education project (in Chinese). Chin J Med Edu. (2021) 41:1029–1032. 10.3760/cma.j.cn115259-20210609-0073530704229

[43] Xianbao S Aiping Y Kehui X. Discussion on further standardization and perfection of continuing medical education in China (in Chinese). Med Res Educ. (2019) 36:67–70. 10.3969/j.issn.1674-490X.2019.02.012

[44] Xuedong L Lilong X Kai F Pengcheng W Sichang H. Analysis on status quo and demand of health professionals' participation in continuing medical education in Sichuan province (in Chinese). Med Soc. (2020) 33:120–4. 10.13723/j.yxysh.2020.10.025

[45] Junyi L. Status quo and thoughts on the continuing medical education program of a general hospital in Shanxi Province in recent years (in Chinese). JingJiShi. (2023) 12:244–245. 10.3969/j.issn.1004-4914.2023.12.116

[46] Zarei M Mojarrab S Bazrafkan L Shokrpour N. The role of continuing medical education programs in promoting Iranian nurses, competency toward noncommunicable diseases, a qualitative content analysis study. BMC Medical Educ. (2022) 22:731:1–14. 10.1186/s12909-022-03804-x 36280836 PMC9589750

[47] Lei S Mingwei C Baozhen L Xiuli X Man X. Investigation and reflection of continuing medical education in primary hospitals in Western China. Northwest Med Edu. (2015) 23:243–5. 10.13555/j.cnki.c.m.e.2015.02.014

[48] Liu JL Mao Y. Continuing medical education and work commitment among rural healthcare workers: a cross-sectional study in 11 western provinces in China. BMJ Open. (2020) 10:e037985. 10.1136/bmjopen-2020-037985 32753451 PMC7406025

[49] Weiguo Q Xiaofei L Zhe Z Yanjun H Jia S Fengge Liu. Study on the cost burden of continuing medical education for medical workers in hospitals in Hebei Province (in Chinese). Chinese Hospital Manag. (2017) 37:53–5.

[50] Grundy Q Millington A Cussen C. Promotion or education: a content analysis of industry-authored oral health educational materials targeted at acute care nurses. BMJ Open. (2020) 10:e040541. 10.1136/bmjopen-2020-040541 33247018 PMC7703418

[51] Grundy Q Cussen C Dale C. Constructing a problem and marketing solutions: A critical content analysis of the nature and function of industry-authored oral health educational materials. J Clin Nurs. (2020) 29:23–4. 10.1111/jocn.15510 32979871

[52] Davis N Davis D Bloch R. Continuing medical education: AMEE Education Guide No 35. Med Teach. (2008) 30:652–66. 10.1080/01421590802108323 18777424

[53] Office of CCMEC. Notice on the application of 2024 national continuing medical education programs. Available at: https://www.cma.org.cn/art/2023/7/12/art_91_51781.html (accessed July 12, 2023).

